# Ultrasound-Assisted Enzyme Extraction, Physicochemical Properties and Antioxidant Activity of Polysaccharides from *Cordyceps militaris* Solid Medium

**DOI:** 10.3390/molecules29194560

**Published:** 2024-09-25

**Authors:** Xiaoya Wang, Jingyan Zhang, Kang Zhang, Zhiting Guo, Guowei Xu, Liping Huang, Lei Wang, Jianxi Li

**Affiliations:** Traditional Chinese Veterinary Technology Innovation Center of Gansu Province, Lanzhou Institute of Husbandry and Pharmaceutical Sciences, Chinese Academy of Agricultural Sciences, Lanzhou 730000, China; wangxy9625@163.com (X.W.);

**Keywords:** *Cordyceps militaris* solid medium, ultrasound-assisted enzyme, polysaccharides, properties, antioxidant

## Abstract

*Cordyceps militaris* solid medium polysaccharides (CMMPs) were extracted using an ultrasound-assisted enzyme method, and the process conditions were optimized via response surface methodology (RSM). The CMMPs were separated into four components named CMMP−1, CMMP−2, CMMP−3 and CMMP−4 using ethanol fractional precipitation, and their monosaccharide composition and structural properties were analyzed by molecular weight analysis, Fourier-transform infrared spectroscopy (FT–IR), scanning electron microscopy (SEM), Congo red test, ultraviolet–visible spectroscopy (UV-vis), atomic force microscopy (AFM), and thermogravimetric analysis (TGA). RSM could predict the yield of the CMMP (R^2^ = 0.9928), and the polysaccharide yield was 15.43% under the selected conditions of 3.1% cellulase enzyme addition, a liquid–solid ratio of 42:1, an extraction temperature of 61 °C, and an extraction time of 60 min. Glucose and galactose were the main constituents of the four fractional precipitated polysaccharides. Furthermore, four components exhibited antioxidant activity, and CMMP−1 demonstrated stronger antioxidant activity in vitro. This study demonstrates the possibility of developing a natural antioxidant food from *Cordyceps militaris* solid medium.

## 1. Introduction

*Cordyceps militaris* solid medium (CMM) is a by-product of the artificial cultivation of *Cordyceps militaris*. It is generally composed of grains, carbon sources, nitrogen sources, vitamins, and mineral elements in proportion to provide nutrients for the production of *Cordyceps militaris. Cordyceps militaris is* an edible mushroom widely produced due to its antioxidant [1], anti-inflammatory [2], and immunomodulation [3] effects. However, significant amounts of CMM are generated, which are typically discarded into the environment when Cordyceps militaris is harvested, with 1 kg of CMM generated for every 1 kg of *Cordyceps militaris* [4], and the CMM also contains mycelium of Cordyceps pupa, which is rich in active ingredients, such as cordycepin, cordyceps acid, and polysaccharides [5], and recycling strategies may be utilized to facilitate the potential innovative application of CMM.

Recently reports demonstrated that polysaccharides from CMM (CMMPs) exhibit anti-tumor [6], antihyperlipidemic, and hepatoprotective activities [7]. However, research on CMMPs has mainly focused on crude polysaccharide extraction and biological activity. The extraction of polysaccharides through water extraction and alcohol precipitation represents a conventional and classical approach in the field [8,9,10], and these are the most commonly used methods for the extraction of CMMPs. Sun et al. adopted the hot reflux water extraction to extract CMMPs, and finally selected an extraction time of 4.5 h at 100 °C [11]. Ren et al. [12] determined that the conditions of extraction were 80 °C and 120 min, but longer extraction time and high temperatures of hot water extraction may result in polysaccharide degradation or even reduce biological activity [13], disadvantages that cannot be ignored. In addition, some novel extraction techniques have been used for CMMPs, including enzyme-assisted [14] and ultrasonic extraction [15]; however, the extraction effect was not ideal. Notably, the combined use of ultrasound and enzymes may improve the extraction efficiency, serving as a suitable method for polysaccharide extraction [16]. The ultrasonic extraction process utilizes the cavitation effect to expedite the destruction of cell walls and enhance the solubilization of bioactive ingredients [17], greatly reducing the extraction time. Meanwhile, the extraction of active ingredients by an enzyme, as a result of the high specificity of the enzyme, can hydrolyze and destroy the cell wall, increasing the release of active ingredients from the intracellular matrix to cause solvent diffusion, with the advantages of mild reaction conditions, environmental protection, and energy savings. Therefore, the ultrasound–enzyme technique has been widely used in polysaccharide extraction [18,19,20], and a report showed that ultrasound–enzyme-extracted polysaccharides of Ginkgo biloba had a unique structure, as well as the strongest antioxidant activity [21]. However, the ultrasonic–enzymatic extraction of CMMPs has rarely been reported.

In this study, to improve the CMMP yield and obtain high efficiency, ultrasound–enzyme-assisted extraction was used; the process parameters were optimized with the RSM, a widely used modeling tool [22], to predict the extraction rate of active ingredients under different extraction conditions [23]. In addition, the different fractions of CMMPs were isolated through ethanol gradient alcohol precipitation. The structural characterizations were analyzed and their antioxidant properties were evaluated in order to be used as a natural antioxidant product to promote recycling and mitigate the environmental impact of waste treatment.

## 2. Results and Discussion

### 2.1. Single-Factor Test

**Figure 1:**
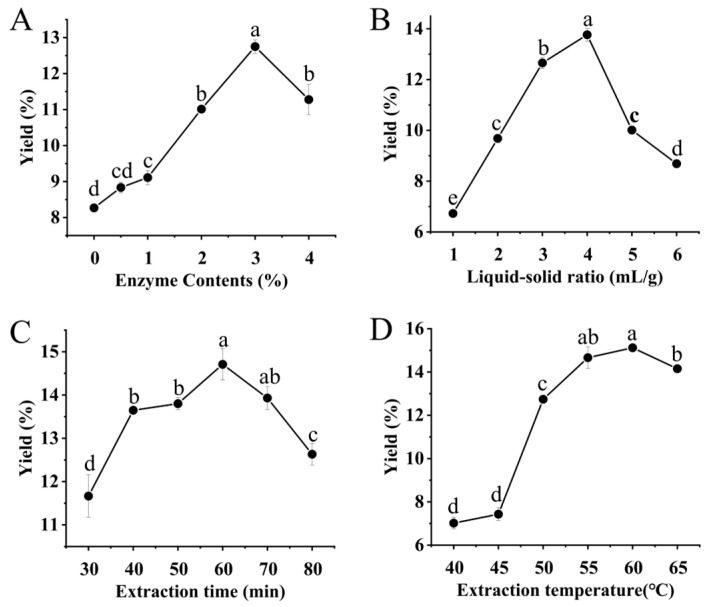
Effect of extraction conditions on CMMP yield: enzyme content (**A**), liquid–solid ratio (**B**), extraction time (**C**), and extraction temperature (**D**) (n = 3). Note: different lowercase letters in the figure indicate significant differences (*p* < 0.05).

The effects of enzyme addition amount, liquid–solid ratio, extraction time, and temperature on the polysaccharide extraction yield were preliminarily determined, with the results presented in Figure 1. According to Figure 1A, with an increase in enzyme addition, the extraction yield demonstrated an increasing trend. The extraction yield reached a peak value of 12.75% with 3% cellulase addition, followed by a subsequent decline, possibly because excessive enzymes degraded a portion of the polysaccharides. Figure 1B shows an initial increase in the liquid–solid ratio, leading to a subsequent enhancement in polysaccharide yield, which we speculated was due to the high proportion of solvent increasing the osmotic pressure of cells and resulting in the outflow of cell material [24]. When the liquid–solid ratio was 40:1, the polysaccharide yield had the highest value, and when the solvent volume was excessively high, the polysaccharide yield decreased, possibly because the internal diffusion distance increased and hydration decreased, which affected mass transfer [25]. And the extraction rate increased (30–60 min), whereas a subsequent decrease was observed over time (Figure 1C). This was possibly due to the instability of the polysaccharide resulting from an excessively prolonged extraction duration, leading to the partial degradation of the polysaccharides [26]. In Figure 1D, we can find that the temperature most suitable for extracting polysaccharides from Cordyceps chrysalis solid medium was 60 °C, and the extraction rate reached 15.12%, indicating that cellulase exhibited the strongest enzyme activity at 60 °C. In addition, excessive temperatures would also reduce the stability of polysaccharide glycoside bonds [27], which could also explain the phenomenon of polysaccharide yield decline when the temperature continued to rise. Therefore, the experiments of the response surface test were designed based on four factors and three levels, and the selected experimental conditions were as follows: enzyme addition amount (2–4%), liquid–solid ratio (30:1–50:1), extraction temperature (55–65 °C), and extraction time (50–70 min).

### 2.2. Extraction Process Optimization

#### 2.2.1. Results of Response Surface

The Box–Behnken design (BBD) was used to optimize the extraction parameters of the CMMPs to improve the polysaccharide yield. A total of 27 test groups with four factors and three levels were designed, and the results demonstrated actual and predicted values (Table 1). On the basis of the test results, multiple regression analysis was performed; the multiple quadratic regression equation (Equation (1)) associated with the variables and response values were obtained as follows:

Y = 15.12 + 0.0208X_1_ + 0.0725X_2_ + 0.4542X_3_ + 0.4642X_4_ − 0.0875X_1_X_2_ + 0.12X_1_X_3_ + 0.845X_1_X_4_ + 0.0225X_2_X_3_ + 0.1825X_2_X_4_ − 0.515X_3_X_4_ − 2.04X_1_^2^ − 0.4333X_2_^2^ − 1.02X_3_^2^ − 1.8X_4_^2^ (1)

The experimental data in Table 1 were analyzed by variance, and the corresponding results are illustrated in Table 2. The *p*-values of the model were significant (*p* < 0.0001), indicating that the equations obtained by this model fit well with the actual values [28], and this model could optimize the ultrasonic enzymatic extraction process of CMMPs. The lack of fit was 0.2636 (*p* > 0.05), suggesting that the error in the experiment was small, and the residual error in the model was caused by random error. In addition, R^2^ = 0.9928 was in an acceptable range, the coefficient of variation (C.V.) was low (1.25), and the difference between the adjusted R^2^ (0.9843) and predicted R^2^ (0.9598) was found to be within a margin of 0.2, indicating high precision and reliability. Adjusted precision was used to measure SNR, which (43.6262) was greater than 4, indicating sufficient signal, and that this model could be used for this extraction process. From the F-values of each primary item in Table 2, we observed that the effect of individual factors on the polysaccharide yield followed liquid–solid ratio > cellulase > extraction time > extraction temperature. The primary terms X_3_ and X_4_ were significant (*p* < 0.0001), indicating that the liquid–solid ratio and the amount of enzyme addition had a significant effect on CMMPs. Interaction terms X_1_X_4_ and X_3_X_4_ were extremely significant (*p* < 0.0001) and X_2_X_4_ was significant (*p* < 0.05), indicating significant interaction between the extraction time and cellulase, liquid–solid ratio and cellulase, and extraction temperature and cellulase. Quadratic terms X_1_^2^, X_2_^2^, X_3_^2^, and X_4_^2^ were extremely significant (*p* < 0.0001), indicating that the influence on CMMP yield was highly significant.

#### 2.2.2. Interaction of Different Factors

**Figure 2:**
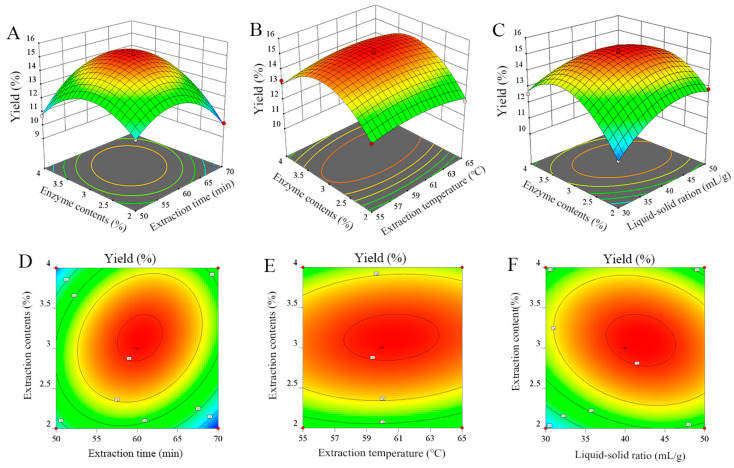
Effect of interaction of two factors on CMMP yield. Three-dimensional charts (**A**–**C**) and corresponding two-dimensional contour curves (**D**–**F**).

The 3D and contour maps of the RSM directly showed the influence of different variables on response value. The opening direction of the surface indicated that the response value, namely the CMMP yield, had a maximum value. Thus, the steeper the curve trend, the stronger the influence of the factor on the yield, while the opposite showed a small influence. Figure 2A shows the highly significant interaction between the extraction time (X_1_) and enzyme addition (X_4_). With an increase in factors X_1_ and X_4_, the yield of CMMPs exhibited an initial upward trend by a subsequent decline. As shown in Figure 2B, CMMPs reached their maximum value with increasing extraction temperature (X_2_) and enzyme addition content (X_4_), when the extraction time (A) and liquid–solid ratio (X_3_) were fixed. After increasing the influencing factors, the CMMP yield started to decrease, and the interaction was significant (*p* = 0.0414 < 0.05). Figure 2C shows that the liquid–solid ratio (X_3_) significantly interacted with the amount of enzyme addition (X_4_) when the extraction time and temperature remained unchanged (*p* < 0.0001). The yield of CMMPs reached a maximum before declining with the increasing liquid–solid ratio and enzyme addition. An ellipse can be observed in the contour maps shown in Figure 2D–F, with the degree of significance positively correlated to the eccentricity of the ellipse. The contour shape reflects the degree of interaction between the two influencing variables [28], and the ellipse indicates significant interaction, while the circle indicates the opposite behavior.

### 2.3. Verification Test

The optimal extraction process optimized by the RSM was as follows: The content of cellulase was 3.109%, the extraction time was 60.314 min, the extraction temperature was 60.536 °C, and the liquid–solid ratio was 41.985:1. The process parameters were adjusted according to actual production, 3.1% cellulase addition, extraction time (60 min), extraction temperature (61 °C), and liquid–solid ratio (42:1). The CMMP yield was 15.43% under these conditions, which closely aligned with the predicted value of 15.195%. This result demonstrates that the optimal process conditions predicted by RSM optimization were accurate and reliable.

### 2.4. Molecular Weight and Chemical Composition

Four polysaccharide components, namely CMMP−1, CMMP−2, CMMP−3, and CMMP−4, were obtained from the ethanol solid culture medium with different ethanol concentrations. The molecular weights of CMMPs are summarized in Table 3. The Mn values of four polysaccharides were 173,376, 60,355, 85,571, and 64,793 Da, the Mw values of the four polysaccharides were 600,804, 65,753, 91,513, and 80,570 Da, and the dispersion coefficients were 3.465, 1.089, 1.069, and 1.243, respectively. The closer the dispersion coefficient was to 1, the more concentrated the molecular weight, which was also the reason for the components’ determination as homogeneous polysaccharides. It can be seen from the data that the molecular weight of CMMP−1 is much higher than that of CMMP−2, CMMP−3, and CMMP−4. Under normal circumstances, low-concentration ethanol tends to precipitate high-molecular-weight polysaccharides, while high-concentration ethanol tends to precipitate low-molecular-weight polysaccharides, but the ethanol concentration does not completely determine the molecular weight of polysaccharides. This also explains why the molecular weight of CMMP−4 is not lower than that of CMMP−2.

### 2.5. Monosaccharide Composition Analysis

**Figure 3:**
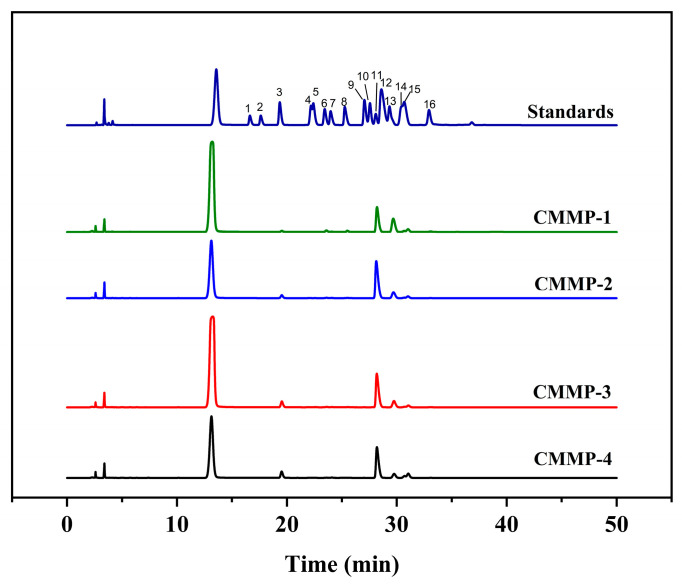
The monosaccharide composition of CMMP−1, CMMP−2, CMMP−3, and CMMP−4. Note: standards: 1 guluronic acid; 2 mannuronic acid; 3 mannose; 4 glucosamine; 5 ribonic; 6 rhamnose; 7 glucuronic acid; 8 galacturonic acid; 9 galactosamine; 10 N-acetylglucosamine; 11 glucose; 12 N-acetylgalactosamine; 13 galactose; 14 xylose; 15 arabinose; 16 fucose.

Figure 3 shows the monosaccharide compositions of the four components. We observed that the four polysaccharides were mainly constituted of glucose and galactose: the glucose content was 72.81%, 86.92%, 81.7%, and 81.33%, and the proportions of galactose were 18.49%, 7.18%, 8.57%, and 5.26%, respectively. In addition, a small amount of 11 monosaccharides was observed, including rhamnose, arabinose, xylose, galacturonic acid, and mannose. The concentration of ethanol could cause changes in the composition ratio of monosaccharides, but did not affect the change in monosaccharide type [29]. The main monosaccharide compositions of the polysaccharides in the medium were found to be comparable to those reported by Wang et al. previously [30], while the monosaccharide composition significantly differed from that of some polysaccharides. This was possibly due to the ultrasonic enzymatic extraction method adopted in this experiment, or the different components retained after purification or ultrafiltration. All of the above circumstances possibly led to the appearance of diverse monosaccharide compositions and molar ratios.

### 2.6. FT-IR Analysis

**Figure 4:**
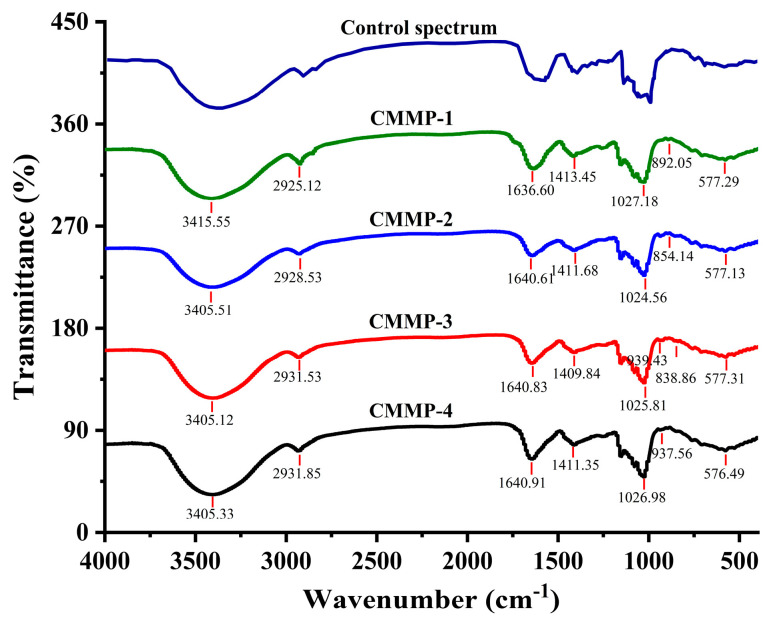
The FT-IR spectra of CMMP−1, CMMP−2, CMMP−3, and CMMP−4, and a control spectrum.

The main functional groups of polysaccharides were identified using FT-IR spectroscopy in this study. Figure 4 presents the infrared spectrum of the CMMP samples, indicating that the absorption peak broad band at 3400 cm^−1^ was due to O-H stretching vibration between the molecules or within the polysaccharide molecules. The weak absorption peaks at 2925–2931 cm^−1^ resulted from the asymmetric C-H stretching vibration of sugars [31], and these two absorption peaks were the characteristic absorption peaks of polysaccharides [32]. The absorption peak in the range of 1636–1640 cm^−1^ was the asymmetric stretching vibration of C=O in COO-, and 1409–1413 cm^−1^ was the characteristic absorption peak of C-H shear vibration. The absorption peak at 1024–1027 cm^−1^ was due to the asymmetric vibrations of C-O-C or C-O-H on the sugar ring, due to the resonance absorption peak of common pyranosaccharide cyclolide and hydroxyl groups, indicating a fragment of pyranosaccharide unit structures [33]. The presence of an alpha-glucoside bond was indicated by the absorption peak observed at 850 cm^−1^ [34].

### 2.7. UV-Vis

**Figure 5:**
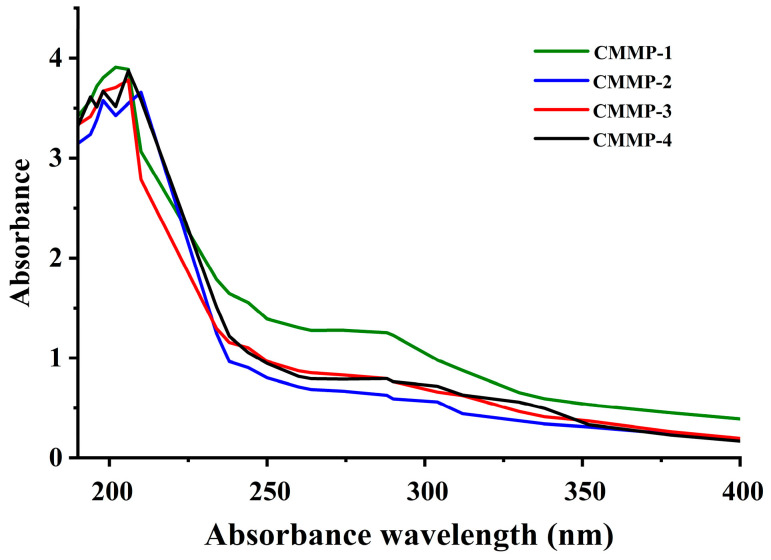
The UV-vis results of CMMP−1, CMMP−2, CMMP−3, and CMMP−4.

The absorption peaks of the polysaccharide samples were mainly observed at 260–280 nm to determine whether protein and nucleic acid impurities were present. As shown in Figure 5, none of the four polysaccharide components were absorbed at 260–280 nm, with no protein impurities, indicating that it was feasible to remove proteins by the Sevag method.

### 2.8. Congo Red Test

**Figure 6:**
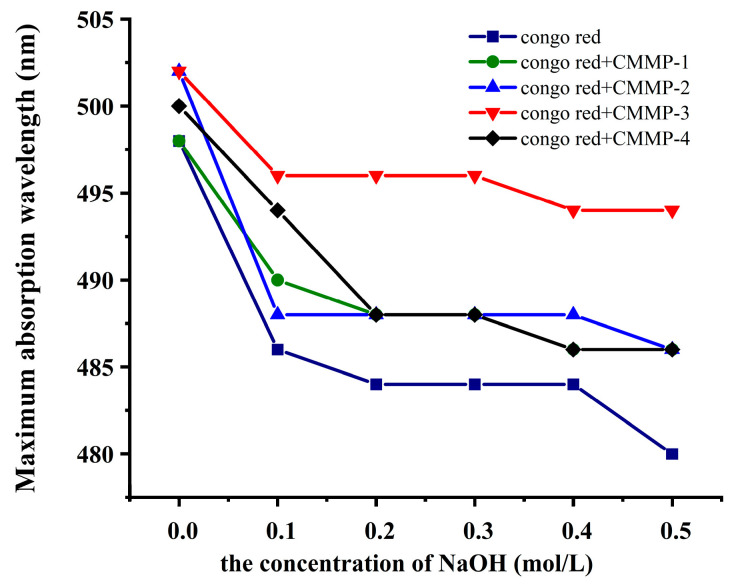
The Congo red test of CMMP−1, CMMP−2, CMMP−3, and CMMP−4.

Certain straight- or branched-chain β-D-glucans demonstrated a triple-helix structure in weakly alkaline or aqueous solutions, and could form complexes with Congo red in a strongly alkaline solution. The maximum absorption wavelength could redshift with an increasing concentration of alkali solution. In this process, the intermolecular and intramolecular hydrogen bonds were destroyed and the triple-helix conformation transformed into a single-chain conformation. When a triple-helix structure was not present in polysaccharides, the change trend exhibited a resemblance to that of Congo red solution. The visible absorption spectrum of the complex decreased with increasing NaOH concentration, as depicted in Figure 6. However, no significant decrease and redshift trend was observed at higher concentrations. The trend of the four components was basically similar to Congo red solution, suggesting that the four CMMP components did not have a triple-helical structure.

### 2.9. SEM

**Figure 7:**
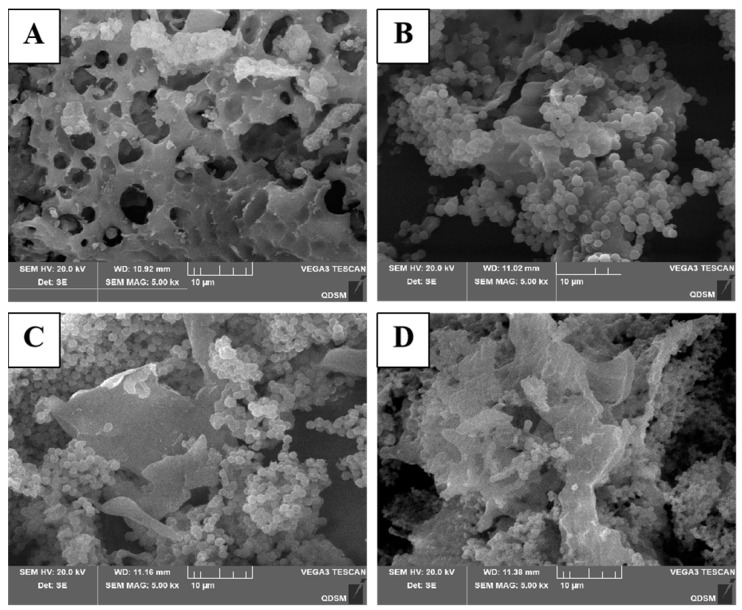
SEM charts (5000×) of CMMP−1 (**A**), CMMP−2 (**B**), CMMP−3 (**C**), and CMMP−4 (**D**).

The microstructures of the CMMPs with four ethanol concentrations were observed by SEM, and the images are presented in Figure 7. Under 5000× magnification, the structure of CMMP−1 was relatively loose, and holes were observed on the surface, indicating that polysaccharide molecules formed a network structure through cross-linking. CMMP−2, CMMP−3, and CMMP−4 were composed of fragmentary and fine granular structures, which may be broken into smaller fragments due to the mechanical oscillation and cavitation of ultrasonic waves extracted by ultrasound. In addition, it is related to their bond energy, aggregation form, and interfacial binding, and the particles in CMMP−2, CMMP−3, and CMMP−4 were observed to be shrinking and separating from each other. This was possibly because polysaccharides with a lower molecular weight could more easily establish hydrogen bonds with water and had difficulty aggregating, demonstrating a relatively dispersed state [35], in which CMMP−2 was more dispersed and its molecular weight was lower.

### 2.10. AFM Analysis

**Figure 8:**
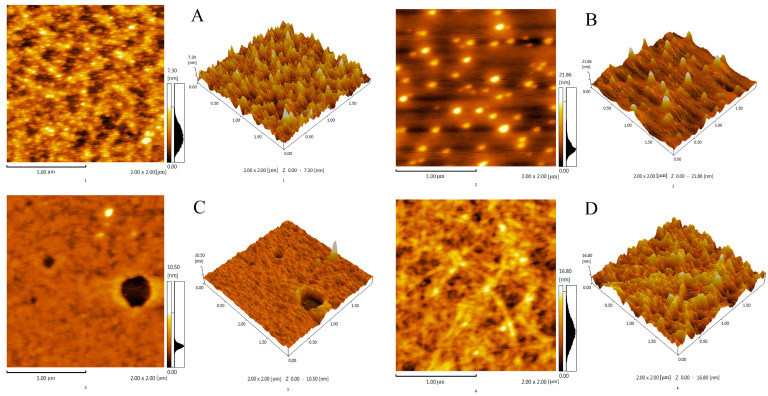
AFM planar image and 3D image of AFM, CMMP−1 (**A**), CMMP−2 (**B**), CMMP−3 (**C**), CMMP−4 (**D**).

AFM serves as a potent tool for the observation of macromolecule surface morphology, providing direct evidence for the chain conformation of polysaccharides [36,37], and has been typically used to identify the rod, linear, and spherical structures of polysaccharides. The AFM pattern of the four polysaccharide components on the mica sheet is shown in Figure 8, with a scanning area of 2 × 2 μm. The morphology and size of the molecular chains of polysaccharides were observed in three dimensions. The four polysaccharides were actually spherical structures with uneven sizes, the particle width of a single polysaccharide was usually 0.1–1 nm [38], and the height of the four polysaccharides reached 7.3, 21.86, 10.5, and 16.8 nm, respectively, indicating that intermolecular or internal interaction aggregation possibly occurred in the polysaccharides. This phenomenon may be caused by the tight binding of the hydroxyl and carboxyl groups of the polysaccharides [39]. When the average polysaccharide diameter was greater than 1, the straight and branched chains of the polysaccharides became entangled with each other to form aggregates.

### 2.11. TGA

**Figure 9:**
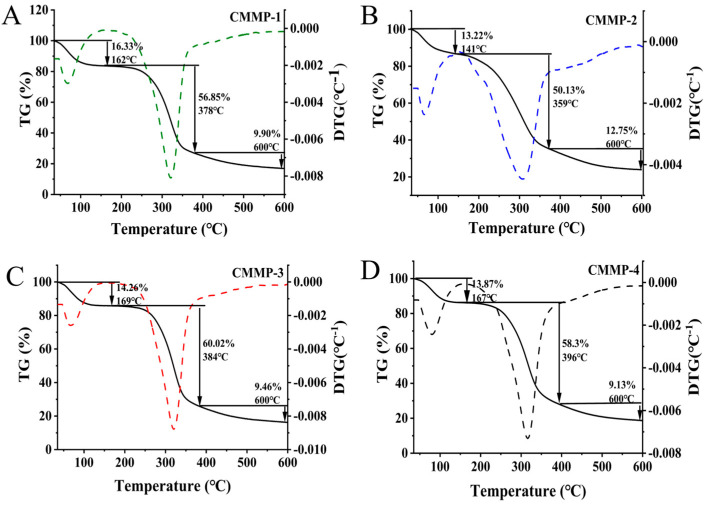
Thermogravimetry results of CMMP−1 (**A**), CMMP−2 (**B**), CMMP−3 (**C**), and CMMP−4 (**D**). The solid line is the TG curve, and the dotted line is the DTG curve.

The TG curve provides valuable information regarding the thermal stability, decomposition temperature, thermal stability temperature range, and substance composition. The TG curve is shown in Figure 9; the thermal stability analysis curve of the four polysaccharides is presented in three stages. The specific initial temperature of mass loss was judged by the TG curve extrapolation method. The TG rate was relatively low, and the main mass loss was due to evaporation loss caused by free water, bound water, and easily decomposed compounds in polysaccharides in the first degradation stage [40]. CMMP−1 showed a loss of 16.33% at 35–162 °C, CMMP−2 showed a loss of 13.22% at 35–141 °C, CMMP−3 had a loss of 14.26% at 35–169 °C, and CMMP−4 had a loss of 13.87% at 35–167 °C.

The second stage involves decomposition caused by the breaking of chemical bonds at high temperatures, with a large number of bond breaks leading to rapid mass loss. The temperature range of the second stage of CMMPs was 162–378 °C, 141–359 °C, 169–384 °C, and 167–396 °C, and the mass loss of CMMPs was 56.85%, 50.13%, 60.02%, and 58.3%, respectively. In the third stage, the mass loss of the four polysaccharides was 9.9%, 12.75%, 9.46%, and 9.13%, respectively. And this stage may be related to the breakdown of the sugar ring structure. The first derivative of the TG curve with respect to temperature was calculated to obtain the derivative thermogravimetry (DTG) curve, which reflected the correlation between the change rate in sample mass and temperature. According to the DTG curve, the main weight loss stage was approximately 200–400 °C, and the maximum weight loss rate temperatures were 321, 304, 320, and 316 °C, respectively. The mass loss of the four polysaccharides at lower temperatures may be related to their structure. More branches of spherical structures are easy to attack, resulting in rapid weight loss.

### 2.12. Antioxidant Assay

To compare the antioxidant capacity of the four polysaccharide components, the antioxidant effect could be achieved by neutralizing free radicals in biological cells; thus, the free radical clearance rate of 2,2′-Azinobis-(3-ethylbenzthiazoline-6-sulphonate (ABTS) assay was performed in this study. The hydroxyl free radical clearance test and total antioxidant capacity (T-AOC, FRAP Method) test have served as the most common methods for evaluating antioxidant capacity [41]. The utilization of these three methodologies has been extensively employed for the assessment of the antioxidant properties exhibited by plants and extracts [42,43].

**Figure 10:**
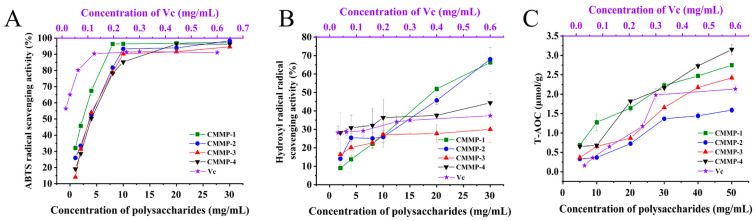
Antioxidant abilities of polysaccharides: ABTS radical scavenging activity (**A**), hydroxyl radical scavenging activity (**B**), T-AOC (**C**).

The ABTS method along with hydroxyl radicals is based on electron transfer [44]. As shown in Figure 10A, CMMP−1 exhibited a strong ABTS radical scavenging capacity when the concentration of the four polysaccharides was identical. When the concentration was in the range of 0–10 mg/mL, the ABTS free radical scavenging ability of the four polysaccharide components exhibited a rapid increase with escalating concentration. The ABTS free radical scavenging ability did not exhibit further enhancement when the concentration was above 10 mg/mL, which was about 90%. The IC50 values of the four polysaccharides were 2.146, 3.222, 3.304, and 3.663 mg/mL, and the IC50 value of Vitamin C (Vc) was 0.007 mg/mL. Therefore, CMMP−1 demonstrated the highest scavenging capacity of the ABTS free radicals, but the value was lower than Vc.

Hydroxyl free radicals are the most powerful oxygen species [24,45]. They possess the ability to readily traverse cellular membranes and interact with a wide range of biomolecules, including proteins, DNA, and lipids. Consequently, they elicit cell demise and contribute to the onset of diverse pathological conditions [46]. The results are presented in Figure 10B, illustrating an increase in the hydroxyl free radical scavenging ability with an elevated concentration range (1–30 mg/mL) of the four polysaccharides. The concentration of polysaccharides exhibited a positive correlation with the rate of hydroxyl radical scavenging, consistent with findings reported by Zhong et al. [47] and Zhang et al. [48]. Obvious enhancement trends of CMMP-1 and CMMP−2 were observed. The scavenging ability of the four polysaccharides was relatively weak.

The T-AOC assay is reflected by the ability to reduce Fe^3+^-tripyridine-triazine (Fe^3+^-TPTZ) to produce blue Fe^2+^-TPTZ in an acidic environment. Figure 10C reveals an increase in the hydroxyl free radical scavenging ability with an elevated concentration range (5–50 mg/mL). CMMP−4 showed the strongest reducing capacity for Fe^3+^, followed by CMMP−1, with values of 3.15 and 2.75 μmol/g, respectively.

## 3. Materials and Methods

### 3.1. Material and Chemicals

The CMM was obtained from Xuzhou Hongyu Agricultural Technology Co., Ltd. (Xuzhou, China), D-(+)-glucose standard was purchased from the China Institute of Food and Drug Certification (Beijing, China), and cellulase (≥50,000 U/g, RY01008) was purchased from Jiangsu Ruiyang Biotechnology Co., Ltd. (Wuxi, China). The ABTS free radical scavenging capacity assay Kit (BC4775), hydroxyl free radical scavenging capacity assay kit (BC1320), and T-AOC assay kit (FRAP Method) (BC1315) were purchased from Solarbio Technology Co., Ltd. (Beijing, China).

### 3.2. Extraction of CMMPs Using the Ultrasonic–Enzymatic Method

The dry CMM was crushed to obtain a powder, and after sieving through 80 mesh, a relatively uniform powder was obtained. First, 1.0 g CMM powder was weighed into the conical bottle precisely, distilled water was added in accordance with the liquid–solid ratio, and a certain amount of enzyme (measured by the mass of substrate) was added. The CMMPs were extracted via combined ultrasonic (600 W) and enzymatic hydrolysis (KQ-600DE, ultrasonic instrument, Kunshan Ultrasonic Instrument Co., Ltd., Kunshan, China). After extraction, the polysaccharides were inactivated at 95 °C for 5 min. The supernatant was obtained by centrifugation to remove residue (4500 rpm, 10 min). Subsequently, crude polysaccharide precipitate was obtained by adding 4 times the volume of anhydrous ethanol and leaving the mixture at 4 °C overnight [49] and dissolved with water. The freeze-dried powder obtained after vacuum freeze-drying (Allegra X-15R, Beckman Coulter Trading Co., Ltd., Shanghai, China) was denoted as crude polysaccharides. After collection and weighing, the CMMPs were obtained, and the sample was dissolved to detect the absorbance by a microplate reader (BIO-TEK Synergy, Winooski, Vermont, USA). The CMMP yield (Y) was calculated using Equation (2).

(2) $$\mathrm{CMMP}\;\mathrm{yield}\;(Y)=\frac{\mathrm{the}\;\mathrm{weight}\;\mathrm{of}\;\mathrm{polysaccharide}}{\mathrm{the}\;\mathrm{weight}\;\mathrm{of}\;Cordyceps\;militaris\;\mathrm{solid}\;\mathrm{medium}}\times 100\%$$

### 3.3. Single-Factor Test

According to the above process, 1.0 g of CMM powder was weighed, cellulase and ultrapure water were added in a certain amount or liquid–solid ratio, and ultrasonic power (600 W) was used for extraction. The enzyme content (0, 0.5%, 1%, 2%, 3%, and 4%, measured by substrate mass), liquid–solid ratio (10:1, 20:1, 30:1, 40:1, 50:1, and 60:1), extraction time (30, 40, 50, 60, 70, and 80 min), and extraction temperature (40, 45, 50, 55, 60, and 65 °C) were investigated, and the specific conditions of the single-factor test are shown in Table 4. Only one variable was changed in each experiment, and the polysaccharide yield under each factor level was used to determine the response surface test range. All tests were performed three times.

### 3.4. RSM Design

The extraction parameter was optimized by BBD. All experiments were designed with 4 factors and 3 levels according to the single-factor assay. The variables consisted of the extraction time (X_1_), extraction temperature (X_2_), liquid–solid ratio (X_3_), and enzyme addition amount (X_4_), which were presented in Table 5. A total of 27 experiments were performed, comprising 24 factorial groups and 3 null groups, with the polysaccharide yield as the response variable. Based on the experimental data, an empirical model was constructed by mathematical modeling and regression analysis, which was a second-order polynomial equation between the response value and the independent variable (Design-Expert 10.0.7 statistical software). The experimental results were fitted by Equation (3):

(3) $$Y=\beta_{0}+\overset{k}{\underset{j=1}{\sum }}\beta_{j}X_{j}+\overset{k}{\underset{j=1}{\sum }}\beta_{jj}X_{j}^{2}+\underset{i}{\sum }\overset{k}{\underset{<j=2}{\sum }}\beta_{ij}X_{i}X_{j}+e_{i}$$

Y: the yield of CMMP (%); *X_i_*, *X_j_*: the coded variables (the value of *i* and *j* ranges from 1 to k); *β*_0_, *β_j_*, *β_jj_*, and *β_ij_* denote the regression coefficients of the intercept coefficient, linear, quadratic, and interaction terms, respectively; *k*: an independent parameter (k = 4); *e_i_*: the error.

**Table 5:** Variables and levels of BBD.

Variables		Level		
		−1	0	1
X_1_	Extraction time (min)	50	60	70
X_2_	Extraction temperature (°C)	55	60	65
X_3_	Liquid–solid ratio (v/m)	30	40	50
X_4_	Enzyme contents (%)	2	3	4

### 3.5. Purification and Characterization of CMMP

#### 3.5.1. Preparation and Purification of CMMP

On the basis of the optimal performing conditions of extraction obtained by the above tests, CMM powder (1 g), 3.1% cellulase (based on the mass of the substrate), and distilled water (1 g/42 mL) were placed in a conical flask, then extracted by ultrasonication at 60 °C for 62 min; the supernatant was collected by centrifugation and precipitated. Then, the precipitate was centrifuged, collected, and dissolved with pure water. Finally, the crude polysaccharides were obtained after protein was removed by the Sevag method. The specific method is to add 1/3 volume of reagent (chloroform: n-butanol = 4:1 (*v*/*v*)) to the sample, stir magnetically for 30 min, centrifuge at 4500 rpm for 15 min, collect the upper liquid (a total of three layers: the middle layer is protein, the lower layer is organic reagent), and repeat the operation until there is no protein layer. The obtained solution was enclosed within a dialysis bag (molecular weight, 3500 Da) [50] and dialyzed by tap water (48 h) and ultrapure water (24 h) [51]. The solution after dialysis was freeze-dried, washed with an appropriate amount of anhydrous ethanol twice, washed with an appropriate amount of acetone twice, washed with an appropriate amount of ether once, and dissolved with an appropriate amount of ultrapure water after the ether volatilized. The crude polysaccharide was stored at −20 °C after freeze-drying.

The ethanol fractional precipitation technology is considered to be a distinctive and convenient approach for producing polysaccharides with significant solubility disparities in alcohols or ketones selectively [52,53]. In this study, a certain amount of CMMP after protein removal and dialysis was added to the distilled water to redissolve. Anhydrous ethanol was gradually added to extraction until the final ethanol concentration was 50% [54], and CMMP−1 was collected after alcohol sedimentation and centrifugation (5000 r/min, 15 min). Then, anhydrous ethanol was added to the supernatant to reach 60% ethanol, and the other process was the same as the above procedure to obtain CMMP−2. Polysaccharide samples CMMP−3 and CMMP−4, precipitated with 70% and 80% ethanol, were also obtained using the same method. Then, the freeze-dried powder of the polysaccharide samples was obtained after freeze-drying and stored at −20 °C.

#### 3.5.2. Molecular Weight Distribution Determination

The polysaccharides obtained after fractional alcohol precipitation (2 mg/mL) were mixed with sodium nitrate (0.1 mol/L), and the determination of the polysaccharides’ molecular weight was performed after 0.22 μm filtration by high-performance gel permeation chromatography (1260 Infinity II MDS, Agilent, Santa Clara, CA, USA).

#### 3.5.3. Monosaccharide Composition Determination

The experimental method for the monosaccharide composition determination of polysaccharides was conducted based on the method by Wang [55]. And the determination of monosaccharide composition was performed using a C18 chromatographic column by HPLC (Agilent 1200, Agilent, Santa Clara, CA, USA).

#### 3.5.4. FT-IR Spectroscopy

After freeze-drying, an appropriate amount of freeze-dried CMMP samples was pressed with dry KBr powder simultaneously, and FT-IR spectroscopy analysis of samples was conducted by an FT-IR spectrometer (Nicolet Is5, Thermo Fisher, Waltham, MA, USA) within the range of 4000 to 400 cm^−1^.

#### 3.5.5. UV-Vis Spectroscopy

The absorption of samples was measured using a UV spectrophotometer (UV-1900, Shimadzu, Tokyo, Japan) at 260 and 280 nm in the range of 190–600 nm.

#### 3.5.6. Congo Red Test

Congo red was used to investigate the triple-helix structure of the CMMPs. The method of this assay was developed by Chen [56]. CMMP−1, CMMP−2, CMMP−3, and CMMP−4 solutions (2 mL, 2.0 mg/mL) were mixed with Congo red solution (2 mL, 80 μmol/L), followed by the addition of a 4 mol/L NaOH solution to the resulting mixture until the final NaOH concentrations reached 0, 0.1, 0.2, 0.3, 0.4, and 0.5 mol/L. The maximum absorption wavelength of samples was recorded in the range of 200–400 nm with a spectrophotometer (EVO300LC, Thermo Fisher, Waltham, MA, USA).

#### 3.5.7. SEM Analysis

The CMMPs were placed on an aluminum plate coated with gold powder through sputtering. SEM images of the CMMPs were obtained using field emission SEM (JSM-840, JEOL, Tokyo, Japan).

#### 3.5.8. AFM

AFM serves as a high-resolution surface imaging technique. In this study, the polysaccharides (1 mg/mL) were dissolved in deionized ultrapure water to the final concentration (0.01 mg/mL). Subsequently, 5 µL of polysaccharide solution (containing 0.01 mg/mL polysaccharide and 0.1 mol /L NH_4_OAc, pH 7) was dropped onto a new mica sheet and air-dried at room temperature (AIST-NT Inc., Novato, CA, USA). AFM observations were then performed after the samples were dried.

#### 3.5.9. TGA

The TGA of four polysaccharides was carried out in a platinum tray under a nitrogen atmosphere using a thermal analyzer (Thermal TGA2, Mettler Toledo, Zurich, Switzerland), and the temperature was raised from 35 °C to 800 °C at a rate of 20 °C/min, and a thermogravimetric curve was drawn with the abscissa as the temperature and the ordinate as the mass.

#### 3.5.10. Comparison of the Antioxidant Capacity

To compare the antioxidant properties of four polysaccharide components, ABTS free radical clearance, hydroxyl free radical clearance, and T-AOC capacity were measured according to the instructions. In the process of determination, the four polysaccharides were prepared into different concentrations of solutions for comparison, with Vc as a positive control.

## 4. Conclusions

The extraction parameters of CMMPs with the ultrasonic–enzymatic method optimized by RSM are as follows: 3.1% enzyme addition, a liquid–solid ratio of 42:1, an extraction temperature of 61 °C, and an extraction time of 60 min. The total polysaccharide yield was 15.43% under this method. Four components (CMMP−1, CMMP−2, CMMP−3, and CMMP−4) were obtained by separating and purifying the total polysaccharides, and their main monosaccharides were glucose and galactose, with characteristic functional groups indicating polysaccharides. The SEM results showed that CMMP−1 had a network structure, and the remaining polysaccharides were composed of fragmented and fine granular structures. The four polysaccharide components had a non-triple-helical structure with a certain stability, according to the Congo red test and TGA. The four polysaccharide components all exhibited antioxidant properties, and CMMP−1 demonstrated stronger antioxidant activity.

The findings may provide a valuable scientific basis for the development of natural antioxidant ingredients from *Cordyceps militaris* solid medium. Further investigations will focus on investigating the antioxidant activity of polysaccharides in vivo and exploring the complex relationship between their structural characteristics and activity.

## Figures and Tables

**Table 1:** Design and results of RSM.

Run	X_1_ (min)	X_2_ (°C)	X_3_ (mL/g)	X_4_ (%)	Actual Yield (%)	Predicted Yield (%)
1	70	60	40:1	2	10.05	9.92
2	60	65	50:1	3	14.28	14.22
3	60	65	40:1	4	13.58	13.58
4	70	60	30:1	3	11.61	11.61
5	60	55	40:1	4	13.29	13.29
6	50	60	30:1	3	11.75	11.75
7	70	60	50:1	3	12.67	12.66
8	60	60	30:1	2	10.77	10.79
9	70	65	40:1	3	12.56	12.66
10	60	60	50:1	2	12.85	12.73
11	60	60	40:1	3	15.13	15.12
12	60	55	40:1	2	12.46	12.46
13	50	55	40:1	3	12.49	12.47
14	60	65	30:1	3	13.38	13.26
15	60	60	40:1	3	15.02	15.12
16	60	55	50:1	3	13.94	14.03
17	60	60	30:1	4	12.56	12.75
18	60	60	40:1	3	15.21	15.12
19	70	60	40:1	4	12.57	12.54
20	50	65	40:1	3	12.88	12.79
21	60	60	50:1	4	12.58	12.63
22	60	55	30:1	3	13.13	13.16
23	50	60	40:1	2	11.57	11.57
24	50	60	50:1	3	12.33	12.38
25	50	60	40:1	4	10.71	10.81
26	60	65	40:1	2	12.02	12.24
27	70	55	40:1	3	12.52	12.69

**Table 2:** Analysis of variance for the results of the BBD.

Source	Sum of Squares	df	Mean Square	F-Value	*p*-Value
Model	41.98	14	3	117.37	<0.0001 **
X_1_—extraction time	0.0052	1	0.0052	0.2039	0.6597
X_2_—extraction temperature	0.0631	1	0.0631	2.47	0.1421
X_3_—solid–liquid ratio	2.48	1	2.48	96.88	<0.0001 **
X_4_—enzyme addition	2.59	1	2.59	101.2	<0.0001 **
X_1_X_2_	0.0306	1	0.0306	1.2	0.2951
X_1_X_3_	0.0576	1	0.0576	2.25	0.1591
X_1_X_4_	2.86	1	2.86	111.79	<0.0001 **
X_2_X_3_	0.002	1	0.002	0.0793	0.7831
X_2_X_4_	0.1332	1	0.1332	5.21	0.0414 *
X_3_X_4_	1.06	1	1.06	41.52	<0.0001 **
X_1_^2^	22.1	1	22.1	865.2	<0.0001 **
X_2_^2^	1	1	1	39.2	<0.0001 **
X_3_^2^	5.53	1	5.53	216.48	<0.0001 **
X_4_^2^	18.72	1	18.72	732.59	<0.0001 **
Residual	0.3066	12	0.0255		
Lack of fit	0.2884	10	0.0288	3.17	0.2636
Pure error	0.0182	2	0.0091		
Cor total	42.29	26			
	R^2^ = 0.9928	R^2^_Adj_ = 0.9843	C.V. = 1.25		

Note: *, *p* < 0.05 and **, *p* < 0.01.

**Table 3:** Molecular weight of each component.

	CMMP−1	CMMP−2	CMMP−3	CMMP−4
Mn (Da)	173,376	60,355	85,571	64,793
Mw (Da)	600,804	65,753	91,513	80,570
Dispersion coefficient	3.465	1.089	1.069	1.243

**Table 4:** Single-factor test design.

Single Factor	Fixed Factors
Enzyme addition (0, 0.5%, 1%, 2%, 3%, 4%)	30:1 v/m, 50 min, 55 °C, 600 W
Liquid–solid ratio (10:1, 20:1, 30:1, 40:1, 50:1, 60:1 v/m)	3%, 50 min, 55 °C, 600 W
Extraction time (30, 40, 50, 60, 70, 80 min)	3%, 40:1 v/m, 55 °C, 600 W
Extraction temperature (40, 45, 50, 55, 60, 65 °C)	3%, 40:1 v/m, 60 min, 600 W

## Data Availability

The data presented in this study are available on request from the corresponding author.
